# In Situ Visible Spectroscopic Daily Monitoring of Senescence of Japanese Maple (*Acer palmatum*) Leaves

**DOI:** 10.3390/life13102030

**Published:** 2023-10-09

**Authors:** Satoru Nakashima, Eri Yamakita

**Affiliations:** 1 Research Institute for Natural Environment, Science and Technology (RINEST), 3-6-32 1F Tarumi-cho, Suita 564-0062, Osaka, Japan; 2 Department of Earth and Space Science, Osaka University, 1-1 Machikaneyama, Toyonaka 560-0043, Osaka, Japan; yamakita.eri.492@m.kyushu-u.ac.jp; 3 Faculty of Agriculture, Kyushu University, 744 Motooka, Nishi-ku, Fukuoka 819-0395, Fukuoka, Japan

**Keywords:** leaf senescence, chlorophylls, anthocyanins, visible spectroscopy, non-destructive daily monitoring, first-order rates, sequential changes, light screen hypothesis

## Abstract

The degradation of green leaves in autumn after their photosynthetic activities is associated with decreases in chlorophylls and increases in anthocyanins. However, the sequential orders of these processes are not well understood because of a lack of continuous monitoring of leaves in the same positions. Therefore, the senescence processes of Japanese maple (*Acer palmatum*) leaves were followed daily in the same positions for approximately 60 days using visible spectroscopy with an original handheld visible–near-infrared spectrometer. The obtained reflection spectra were converted to absorption spectra and band areas of chlorophyll a and anthocyanins were determined. Decreases in the chlorophyll a band area with time show two-step exponential decreases corresponding to slow and fast first-order decrease rates. A rapid decrease in chlorophyll a started after an increase in anthocyanin. Therefore, the leaf senescence started through a slow decrease in chlorophyll a (20–30 days), followed by a rapid increase in anthocyanins (~20 days), followed by a rapid decrease in chlorophyll a (10–20 days). The formation of anthocyanins has been proposed to protect leaf cells from losing chlorophylls through solar radiation damage. The obtained sequential changes of pigments support this light screen hypothesis. (199 words < 200 words)

## 1. Introduction

**Figure 1:**
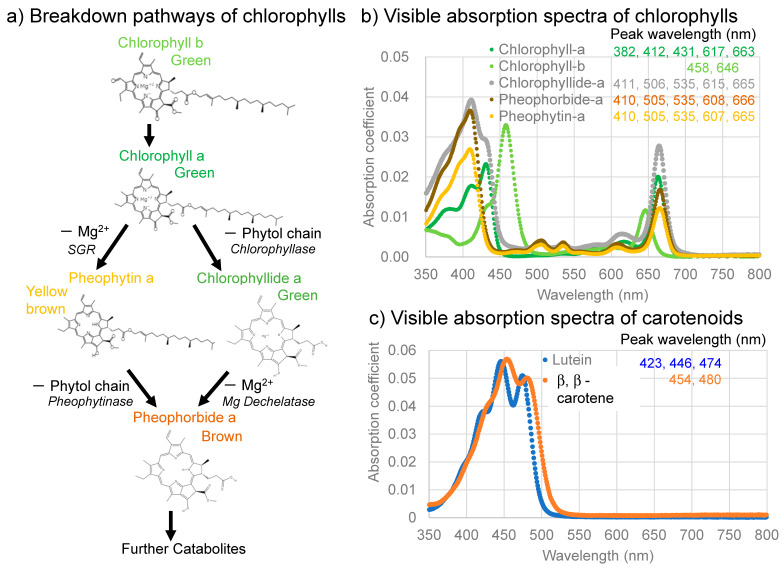
(**a**) Two primary breakdown pathways of chlorophylls with their colors: left, via pheophytin a, and right, via chlorophyllide a with possible corresponding enzymes; (**b**) Visible absorption spectra of chlorophylls and their breakdown products (chlorophyll a, b, chlorophyllide a, pheophorbide a, and pheophytin a (in 90% acetone)); (**c**) Visible absorption spectra of representative carotenoids (lutein in 100% ethanol and β, β-carotene in 100% acetone). These spectra are drawn from spectral data of commercially available pigment standards from [6].

Photosynthesis of plants is the primary production process changing sunlight and CO_2_ to sugars, ATP, and NADP, as well as O_2_, which supports all the activities of living organisms on Earth [1,2]. Photosynthesis occurs in the thylakoid membranes of chloroplasts by means of several embedded proteins, including photosynthetic pigments such as chlorophylls. In oxygen-producing photosynthesis, a combination of photosystem I (PSI) and photosystem II (PSII) is used for the transfer of electrons and protons. Chlorophyll a (Figure 1a,b) is the primary antenna pigment accepting sunlight around 680–700 nm [1,2].

The photosynthetic activities of plants generally increase from spring to summer, then decrease from autumn to winter. These changes correspond to increases and decreases in chlorophyll a during these periods [3]. The biosynthesis of chlorophylls generally occurs in the chloroplasts by means of a complex series of chemical reactions, beginning with the conversion of L-glutamic acid to protoporphyrin, which is then converted via chlorophyllide to chlorophyll a and b [1,2,4]. Therefore, the decomposition pathways of chlorophyll a have been traditionally considered to proceed from chlorophyll a (green), via chlorophyllide a (green) or chlorophyllin a (light green), to pheophorbide a (brown) through a reversal in the biosynthetic pathway [2,5] (Figure 1a, right side). The visible absorption spectra of these chlorophyll molecules are shown in Figure 1b.

On the other hand, the breakdown of chlorophylls in vegetables and fruits during the processing and heating of plants have been extensively studied in food and cooking sciences [7,8,9,10,11]. In these studies, chlorophyll a lost Mg^2+^ in its tetrapyrrole ring to pheophytin a (yellow brown), then changed, via an ester decomposition eliminating a phytol chain, to pheophorbide a (brown) (Figure 1a, left side); the chlorophyllide/chlorophyllin (green) pathway might also occur under certain processing conditions (Figure 1a, right side).

Recent studies indicated a natural chlorophyll a breakdown pathway via pheophytin in some plants (Figure 1a, left side) [12,13,14,15,16,17,18,19]. Schenk et al. [12] observed degradation of the green color of *Arabidopsis* by deactivating chlorophyllase, while Schelbert et al. [14] found that pheophytinase also contributes to the degradation of the green color of *Arabidopsis*. Azoulay Shemer et al. [13] also found that pheophytinase drives the degradation of the green color of lemon fruits. These studies indicated that the removal of the phytol chain of chlorophyll a by chlorophyllase, forming chlorophyllide, does not generally occur (Figure 1a, right side), while pheophytinase drives the removal of Mg^2+^ from chlorophyll a, forming pheophytin, in some natural plant systems (Figure 1a, left side). Moreover, STAY-GREEN (SGR) proteins have been reported to destabilize chlorophyll–protein complexes, spurring the further degradation of chlorophylls and proteins [2,20,21,22]. Therefore, the assessment of chlorophyll a breakdown processes in nature remains complex and requires further detailed studies.

In the late autumn, the senescence of the green leaves of several plants, such as Japanese maple trees (*Acer palmatum*), is particularly pronounced due to color changes in the leaves from green to red or yellow [23,24]. The color change from green to yellow in Japanese maple leaves corresponds to decreases in chlorophylls (green) and the persistence of carotenoids (yellow) (Figure 1c). On the other hand, the color change from green to red in Japanese maple leaves corresponds to decreases in chlorophylls (green) and increases in anthocyanins (red). The red-senescing leaves of red-osier dogwood (*Cornus stolonifera*), which contain anthocyanins, were considered to reduce the risks of photo-oxidative damage by bluish sunlight [25]. Based on these observations, the light screen hypothesis has been proposed for red leaf senescence, which posits that the formation of anthocyanins (red pigment) is for the protection of leaf cells losing chlorophylls against the damages of sunlight [26,27]. Although several studies quantified decreases and increases in chlorophylls, anthocyanins, and carotenoids in senescing leaves by extracting these pigments in solvents from representative samples [23], no detailed sequential changes with time in these pigments have been reported.

Direct non-destructive measurements of reflection spectra of plant leaves have been conducted and several reflectance indices based on the reciprocals of reflectance (R) values (1/R) in several wavelength regions have been proposed to estimate the contents of pigments such as chlorophylls, carotenoids, and anthocyanins for the remote sensing of plants [28,29,30,31,32]. These reflectance indices were found to be well-correlated with the contents of chlorophylls, carotenoids, and anthocyanins in some plant leaves. However, in spectroscopy, concentrations of materials c (mol·L^−1^) are proportional to absorbance (Abs) according to Lambert–Beer’s law:

Abs = −log R = log (1/R) = ε c l (1)

where ε is the molar absorption coefficient (L·mol^−1^·cm^−1^) and l is the sample thickness (cm).

Since the sample thickness variations and the absolute reflectance values compared to a reference, such as a white plate, cannot be obtained through remote sensing of plant leaves and the textures of plant leaves also affect the reflectance values, reflectance indices based on 1/R rather than log (1/R) have been proposed and employed in non-destructive determinations of pigment contents in plant leaves. This is a kind of approximation of an exponential curve by a linear trend at certain ranges of lower concentrations. Therefore, the reflectance indices have some limitations in principle.

On the other hand, in absorption spectroscopy, absorbance (Abs) values at the characteristic peak wavelengths of pigments in leaves extracted in solvents are used to determine pigment concentrations based on Lambert–Beer’s law (Equation (1)), generally with no absorbance of solvents. However, for direct absorption spectroscopy of plant leaves, simple absorbance at a certain wavelength cannot be used, since absorption bands are not from zero baselines but from surrounding absorption bands of coexisting pigments and certain internal structures of leaves. Therefore, the absorbance of a selected pigment should be determined principally through the band areas of its absorption bands, with appropriate baselines avoiding overlapping bands of other species. This kind of method was recently applied to the ripening process of a mini tomato to evaluate changes in the band areas of chlorophyll a, carotenoids, and lycopene with time [33]. Although the absolute contents of these pigments could not be determined because chemical analyses were impossible for the same mini tomato sample during its ripening process, relative changes in these band areas with time indicated relative changes in these pigments’ contents, which could be used to determine the rate constants of their changes [33].

In this study, the senescence processes of Japanese maple (*Acer palmatum*) leaves were followed daily in the same positions for three periods (in 2022–2023, 2021–2022, and 2016) through visible spectroscopy using an original handheld visible spectrometer covering wide spectral ranges (400–1050 nm) with a sufficient spectral resolution, capturing spectral changes in detail. Decreases and increases in chlorophylls and anthocyanins with time were quantitatively described by their absorption band area changes and compared with the results calculated from their reflectance indices. The obtained sequential changes in these pigments were used to examine chlorophyll breakdown pathways and the sunlight screen hypothesis of leaf senescence. Although the absolute pigment contents could not be obtained because of the impossibility of conducting multiple chemical analyses of the same samples during the senescence, the relative changes in the band areas were used to determine the first-order decrease rate constants of chlorophyll a in (s^−1^) without concentration terms in their units.

## 2. Materials and Methods

### 2.1. Materials

A commercially available young Japanese maple (*Acer palmatum*) (a variety named Katsura) was purchased and planted in a planter set on the veranda of the first author’s home in Osaka, Japan. The young tree was grown on the veranda for 65 days (from 31 October 2022 to 4 January 2023) and was watered every day. No fertilizer was added.

The second series of daily monitoring of another Japanese maple (Momiji) leaf took place from 14 November 2021 to 11 January 2022 (59 days) on the veranda of the first author’s home. Data are lacking from the fourth to eighth days because the light source of the spectrometer was being changed.

The third series of daily monitoring of Japanese maple (Momiji) leaves was conducted by the second author from 24 October 2016 to 25 November 2016 (32 days) on four leaves of a naturally grown maple tree on the campus of Osaka University.

### 2.2. Handheld Visible–Near-Infrared Spectrometer

**Figure 2:**
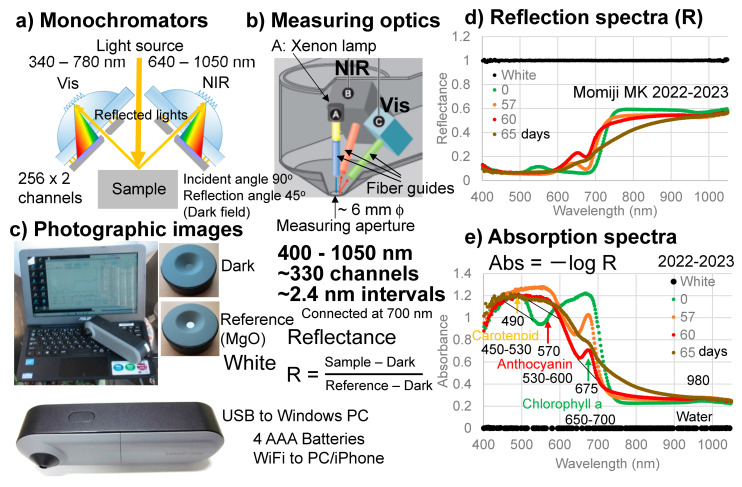
Handheld visible–near-infrared spectrometer. (**a**) Schematic configuration of the two monochromators; (**b**) Measuring optics; (**c**) Photographic images of the spectrometer and the dark and white cover plates; (**d**) Reflection spectra of the white reference (MgO) and a Japanese maple (Momiji) leaf at 0, 57, 60, and 65 days; (**e**) Absorption spectra of the white reference (MgO) and the Japanese maple (Momiji) leaf at 0, 57, 60, and 65 days. The band maxima wavelengths (nm) and linear baseline regions for determining band areas are indicated for carotenoids, anthocyanins, and chlorophyll a.

An original handheld visible–near-infrared spectrometer (Mirage Cross, Fuso Precision, Kyoto, Japan) was developed for this study (Figure 2). The details of this instrument were reported separately [33]. The spectrometer can cover wavelength regions from 400 to 1050 nm by using two monochromators with about 330 channels at approximately 2.4 nm intervals. A xenon (Xe) lamp was applied to the sample surface using a fiber guide and reflected lights from the sample were collected through fiber guides at 45 degree angles (Figure 2a,b).

A dark signal was first measured without the light source by using an original cover plate covering the measuring aperture, which was approximately 6 mm in diameter. Then, a reference signal was measured with the light source by using another face of the original plate with a white reference made of MgO (Figure 2c). Reflected light intensities from a sample were obtained using the following equation:

Reflectance R = (Sample − Dark)/(Reference − Dark) (2)

The reflection spectrum of the white reference composed of MgO measured by this spectrometer is shown in Figure 2d. By taking Abs = −log R, its absorption spectrum can be obtained as shown in Figure 2e. Measurement of a sample takes approximately 3 s.

The advantages of this original instrument include: (1) covering a wide wavelength range (400–1050 nm) with sufficient spectral resolution, enabling the detailed detection of spectral band features; (2) handheld and easy operation without fibers, suitable for onsite field uses; (3) easy direct spectral measurements connected to a notebook PC supplying electricity to the spectrometer; and (4) capability of using commercial batteries for spectrometers and transmission of data acquisitions to iPhones via WiFi [33]. These enabled onsite daily spectral monitoring of the senescing leaves of Japanese maples.

### 2.3. Daily Measurements of the Japanese Maple Leaves

**Figure 3:**
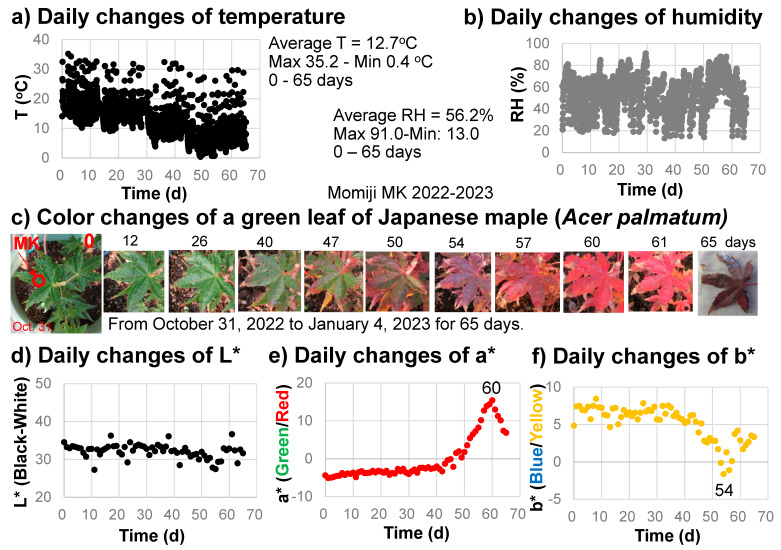
Daily monitoring of the senescing processes of a Japanese maple leaf from 31 October 2022 to 4 January 2023 (65 days). (**a**) Temperature changes with time; (**b**) Relative humidity (RH) changes with time; (**c**) Photographs of the leaf with color changes; (**d**) L* value (black/white) changes with time; (**e**) a* value (green/red) changes with time; (**f**) b* value (blue/yellow) changes with time.

Three green leaves of Japanese maple (*Acer palmatum*) (a variety of Momiji named Katsura) were selected to monitor their color changes every day for 65 days (from 31 October 2022 to 4 January 2023) (Figure 3). The same positions (Figure 3c) were measured by the spectrometer daily at around 8 a.m. A temperature, humidity, and illuminance sensor (Sanwa supply TPHU5, Okayama, Japan) was set near the sample and these values were monitored every hour. One of the leaves became dirty and another fell; consequently, only one representative series of measurements showing systematic color changes is presented.

The same procedure was conducted for two leaves of Japanese maple (Momiji) in the same positions from 14 November 2021 to 11 January 2022 (59 days); only one representative series is presented.

Four leaves of a naturally grown maple (Momiji) tree on the campus of Osaka University were measured in the same positions by the second author using another original handheld visible spectrometer (400–760 nm) [34] from 24 October 2016 to 25 November 2016 (32 days). Among these data, triplicate measurements of the same position of a leaf are presented.

### 2.4. Determination of L*a*b* Color Values (Second CIE, 1976)

Colors of the sample leaves are represented in this study in the L*a*b* color space recommended by the Commission Internationale d’Eclairage (CIE) (second CIELab 1976 color space) [35,36]. L*, a*, and b* values were calculated using CIE XYZ tristimulus values from reflectance spectra in the 400 to 700 nm range with 10 nm intervals according to the following procedures.

(3) $$X=k\int S\left(\lambda \right)R\left(\lambda \right)\bar{x}\left(\lambda \right)$$

(4) $$Y=k\int S\left(\lambda \right)R\left(\lambda \right)\bar{y}\left(\lambda \right)$$

(5) $$Z=k\int S\left(\lambda \right)R\left(\lambda \right)\bar{z}\left(\lambda \right)$$

Here, $S\left(\lambda \right)$ is a spectral distribution of the illuminating light source, $R\left(\lambda \right)$ is the sample reflectance spectrum, $\bar{x}\left(\lambda \right)$, $\bar{y}\left(\lambda \right)$, and $\bar{z}\left(\lambda \right)$ are the color-matching functions, and k is the normalization constant given by k = 1/[$\int S\left(\lambda \right)R\left(\lambda \right)\bar{x}\left(\lambda \right)$]. L*, a*, and b* values were calculated using the following equations:

(6) L*=116(YYn)13−16

(7) a*=500(XXn)13−(YYn)13

(8) b*=200(YYn)13−(ZZn)13

where X_n_, Y_n_, and Z_n_ are the tristimulus values of the nominally white objective color stimulus. These equations had the constraints that X = X_n_, Y = Y_n_, and Z = Z_n_ > 0.01.

L* is lightness, with 0 and 100 corresponding to black and white, respectively. Negative a* values correspond to green and positive a* values correspond to red. Negative b* values correspond to blue and positive b* values correspond to yellow. These L*a*b* values are widely used in food, agricultural, and material sciences for quantitatively describing the colors of objects and materials [7,8,9,10,11,33,34,35,36].

### 2.5. Determination of Reflectance Indices and Contents of Chlorophylls and Anthocyanins

The following reflectance indices, often used for the remote sensing of plants [28,29,30,31,32], were calculated for the Japanese maple leaves using reflectance (R) values around 550 nm (green) (R_550_), 700 nm (red edge) (R_700_), and 790 nm (NIR) (R_790_) for chlorophylls (Chl); around 510 nm (R_510_), 570 nm (green) (R_570_), 700 nm (red edge) (R_700_), and 790 nm (NIR) (R_790_) for carotenoids (Car); and around 570 nm (green) (R_570_), 700 nm (red edge) (R_700_), and 790 nm (NIR) (R_790_) for anthocyanins (Anth):

Chl green index (CI_green_) = (1/R_550_ − 1/R_790_) × R_790_ = R_790_/R_550_ − 1 (9)

Chl red-edge index (CI_red edge_) = (1/R_700_ − 1/R_790_) × R_790_ = R_790_/R_700_ − 1 (10)

Car green index (Car_green_)= (1/R_510_ − 1/R_570_) × R_790_ = R_790_/R_510_ − R_790_/R_570_ (11)

Car red-edge index (Car_red edge_) = (1/R_510_ − 1/R_700_) × R_790_ = R_790_/R_510_ − R_790_/R_700_ (12)

Anth red-edge index (mARI) = (1/R_570_ − 1/R_700_) × R_790_ = R_790_/R_570_ − R_790_/R_700_ (13)

Total chlorophyll contents (nmol·cm^−2^) were calculated from the Chl red-edge index (CI_red edge_) using the following calibration equation [32]:

Chl content (nmol·cm^−2^) = (Chl red edge − 0.0319)/0.0737 (14)

Total anthocyanin contents (nmol·cm^−2^) were calculated from the Anth red-edge index (mARI) using the following calibration equation [32]:

Anth content (nmol·cm^−2^) = ln {1 − (Anth red edge/13.73)}/(−0.018) (15)

### 2.6. Determination of Absorption Spectra and Band Areas

Although diffuse reflectance values can be used as quantitative spectra for granular or powdery thick materials, thin leaf samples are not considered to be composed of granular structures with diffuse reflection. Moreover, negative logarithm of reflectance (absorbance) values are often taken as quantitative spectra in reflection spectroscopy of plants and food products [37,38,39,40]. Therefore, in this study, reflectance (R) values were converted to absorbance (Abs) values using Equation (1) (Abs = −log R). This absorbance (Abs) is directly related to the concentrations (c) (mol· l^−1^) of pigments in visible spectroscopy through Lambert–Beer’s law (Equation (1)). Representative reflection (reflectance R) spectra of the sample leaf (Figure 2d) are converted to corresponding absorption (absorbance) spectra in Figure 2e.

As the absorption (absorbance) spectra in Figure 1b,c and Figure 2e show, absorption bands overlap among chlorophyll a and b, carotenoids, and anthocyanins, meaning the absorbance at a single wavelength cannot be representative of each pigment. Therefore, to examine the quantitative changes in the absorption bands observed in the absorption spectra of the sample leaves over time, the following band areas were determined (Figure 2e).

The band areas around 490 nm caused by carotenoids and chlorophylls were determined using a linear baseline from 450 to 530 nm to avoid overlaps with other bands in the shorter and longer wavelength regions (Figure 2e). They were divided by their initial values to obtain dimensionless normalized band areas used to represent their changes with time.

The band areas around 570 nm caused by anthocyanins were determined using a linear baseline from 530 to 600 nm to avoid overlaps with the bands of carotenoids and chlorophylls (Figure 2e). They were divided by their maximum values to obtain dimensionless normalized band areas used to represent their changes with time.

The band areas around 675 nm caused mainly by chlorophyll a were determined using a linear baseline from 650 to 700 nm to avoid overlaps with the bands of anthocyanins and chlorophyll b (Figure 1b and Figure 2e). Since this narrow region corresponds mainly to the absorption band of chlorophyll a, with only small contributions from chlorophyll b [1,6], this band was taken as the chlorophyll a (Chl a) band. The band areas were divided by their initial values to obtain dimensionless normalized band areas used to represent their changes with time.

The band areas around 980 nm due to water and sugars were not determined because they are very small (Figure 2e).

The assignments of these bands and their shifts will be discussed later.

## 3. Results

### 3.1. Temperature, Humidity, and Luminance

The temperature and relative humidity values during the monitoring period from 31 October 2022 to 4 January 2023 (65 days) are shown in Figure 3a,b. The temperature ranged from 0.4 to 35.2 °C, with an average value of 12.7 °C. The relative humidity ranged from 13.0 to 91.0%, with an average value of 56.2%. The illuminance ranged from 0 to approximately 18,000 lux. The sample position on the veranda, oriented toward the southeast, received direct sunlight in the morning from about 7 to 9 a.m.

### 3.2. Color Changes

The daily changes in the L*a*b* color values calculated from the reflection spectra of the sample leaf in 2022–2023 are shown in Figure 3d–f.

The L* values (black–white) decreased slightly from about 35 to 30 (becoming blackish) during the leaf color change period (Figure 3d). The a* values (green/red) increased slightly from −5 to –3 (0–40 days), staying green, then increased rapidly, attaining their maximum of +15.4 at 60 days (reddest) before finally decreasing to approximately 7 when the brown leaf fell (Figure 3c,e). The b* values (blue/yellow) decreased slightly, from +7 to +5, during the first 40 days, then decreased rapidly to −1.6 at 54 days before finally increasing back to +3 at 65 days (Figure 3f).

These data on daily color changes in the sample leaf clearly show the continuous color change processes during autumn senescence due to daily changes in green (−a* value), red (+a* value), and yellow (+b* value) components in the leaf.

### 3.3. Reflection and Absorption Spectral Changes

Representative reflection (reflectance R) spectra of the sample leaf in 2022–2023 are shown in Figure 2d. They were converted to absorption spectra by taking the absorbance (Abs) = −log R, shown in Figure 2e.

The initial green leaf showed broad absorption bands in the 550–750 nm region centered around 675 nm. This 675 nm band is an origin of the green color of the leaf and is considered to be mainly due to chlorophyll a, since a 670–680 nm band was generally reported in direct spectroscopic measurements on plants including leaves [41,42,43,44,45]. However, the absorption maxima of chlorophyll a in organic solvents such as diethyl ether and acetone are reported to be around 382, 412, 431, 617, and 663 nm, with the longest band around 660 nm [1,6,46] (Figure 1b). Possible band shifts of these bands will be discussed later.

The broad band around 675 nm can also include contributions at the shorter wavelength region around 646 nm from chlorophyll b [1,6,47] (Figure 1b), which will be discussed later.

Another broad band in the 400–550 nm region, centered around the 490 nm band, is the origin of the yellowish color of the leaf. This broad band around 490 nm can be due mainly to carotenoids such as β-carotene, with several absorption maxima around 454 and 480 nm [6,47] (Figure 1c) and contributions from chlorophyll a around 430 nm and from chlorophyll b around 460 nm [6,41] (Figure 1b). This will be discussed later.

The red leaves at 57 and 60 days showed clear decreases in the 675 nm band, primarily due to chlorophyll a (Figure 2e). This 675 nm band became minimal for the brown leaf at 65 days (Figure 2e).

On the other hand, a broad band around 570 nm increased greatly for red leaves (Figure 2e). This 570 nm band can be assigned to anthocyanin [47]. However, the band maximum position is shifted to the longer wavelength region from the reported band position extracted in organic solvents, centered around 546 nm [47]. This will be discussed later.

### 3.4. Changes with Time in Band Areas

**Figure 4:**
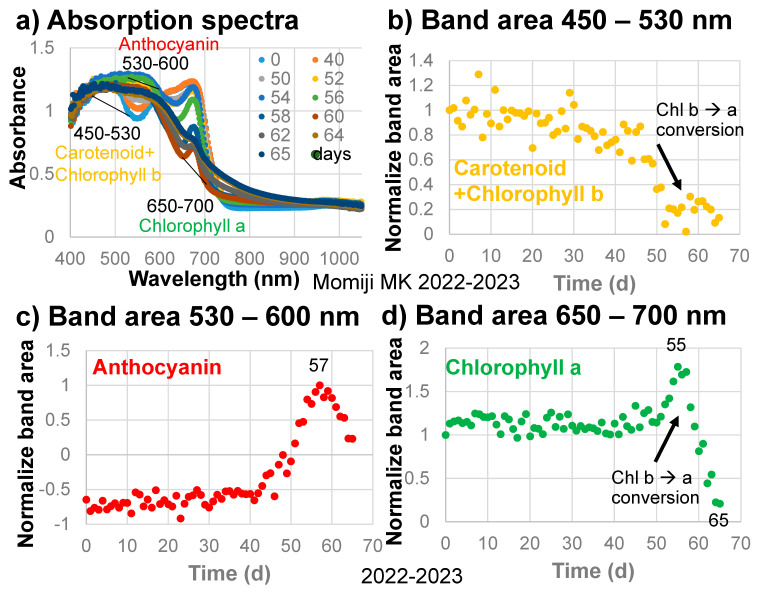
(**a**) Changes in the absorption spectra of the Japanese maple leaf with time, with baseline regions for determining the band areas of carotenoids and chlorophyll b, anthocyanins, and chlorophyll a; (**b**) Changes in the normalized band areas of carotenoids and chlorophyll b (450–530 nm) with time; (**c**) Changes in the normalized band areas of anthocyanins (530–600 nm) with time; (**d**) Changes in the normalized band areas of chlorophyll a (650–700 nm) during the daily monitoring of the senescing processes of a Japanese maple leaf from 31 October 2022 to 4 January 2023 (65 days).

The band areas around 490 nm, with a linear baseline from 450 to 530 nm for carotenoids and chlorophylls (Figure 4a), divided by their initial values are plotted against time in Figure 4b for the sample leaf in 2022–2023. The 450–530 nm band area of carotenoids and chlorophylls decreased gradually throughout the leaf senescence period of 65 days (Figure 4b).

The band areas around 570 nm, with a linear baseline from 530 to 600 nm for anthocyanins (Figure 4a), divided by their maximum values are plotted against time in Figure 4c. The 530–600 nm band area of anthocyanins increased slightly from 0 to 40 days but remained negative because of the presence of the carotenoid and chlorophyll b band and the chlorophyll a band on either side (Figure 4c). The anthocyanin band area then increased rapidly up to day 57, but decreased rapidly up to day 65 in the final browning stage. These changes in the anthocyanin band are very similar to changes in the a* values (green/red) with time (Figure 3e). Therefore, the anthocyanin band is considered to control the reddish color of the sample leaf.

The band areas around 675 nm, due mainly to chlorophyll a, with a linear baseline from 650 to 700 nm (Figure 4a), divided by their initial values are plotted against time in Figure 4d. The 650–700 nm band area of chlorophyll a decreased slightly from 0 to 40 days, then increased until day 55, then finally decreased rapidly up to day 65 (Figure 4d). This Chl a decrease will be discussed and analyzed later.

### 3.5. Changes in Reflectance Indices with Time

**Figure 5:**
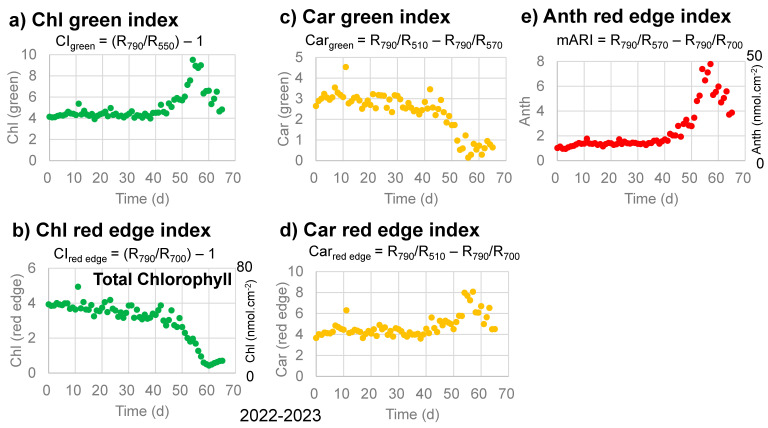
Changes in reflectance indices with time, as proposed by Gitelson et al. (2006; 2009) [31,32], during the daily monitoring of the senescing processes of a Japanese maple leaf from 31 October 2022 to 4 January 2023 (65 days). (**a**) Chlorophyll green index (CI_green_); (**b**) Chlorophyll red-edge index (CI_red edge_) with corresponding total chlorophyll contents (nmol·cm^−2^) on the right vertical axis; (**c**) Carotenoid green index (Car_green_); (**d**) Carotenoid red-edge index (Car_red edge_); (**e**) Anthocyanin red-edge index (mARI) with corresponding anthocyanin contents (nmol·cm^−2^) on the right vertical axis.

Changes in the Chl green index (CI_green_) and Chl red-edge index (CI_red edge_) with time for chlorophylls [30,31,32] are plotted in Figure 5a,b for the sample leaf in 2022–2023. The Chl green index (CI_green_) was relatively stable until day 40, increased until day 55, then decreased back to the initial level (Figure 5a). On the other hand, the Chl red-edge index (CI_red edge_) decreased slowly from 0 to 40 days, then rapidly to day 60. These trends are different from the band area (650–700 nm) for chlorophyll a in Figure 4d and will be discussed later. If we calculate total chlorophyll contents (nmol·cm^−2^) from the Chl red-edge index (CI_red edge_) using the calibration equation (Equation (14)) [32], the total chlorophyll contents decreased from approximately 53 to approximately 6 nmol·cm^−2^ during the senescence of the sample leaf in 2022–2023.

Changes in the Car green index (Car_green_) and Car red-edge index (Car_red edge_) with time for carotenoids [31,32] are plotted in Figure 5c,d for the sample leaf in 2022–2023. The Car green index (Car_green_) decreased slowly until day 40, then rapidly until day 55 (Figure 5c). This trend is similar to the 450–530 nm band area for carotenoids in Figure 4b. On the other hand, the Car red-edge index (Car_red edge_) stayed relatively stable until day 40, increased until day 55, then decreased. These trends are different from the band area (450–530 nm) for carotenoids in Figure 4b and will be discussed later.

Changes in the Anth red-edge index (mARI) with time for anthocyanins [31,32] are plotted in Figure 5e for the sample leaf in 2022–2023. The mARI values were relatively stable until day 40, increased until day 55, then decreased (Figure 5e). These trends are quite similar to those of the band area (530–600 nm) for anthocyanins in Figure 4c and will be discussed later. If we calculate anthocyanin contents (nmol·cm^−2^) from the Anth red-edge index (mARI) using the calibration equation (Equation (15)) [32], the anthocyanin contents (nmol·cm^−2^) increased from approximately 6 to approximately 50, then decreased to approximately 25 nmol·cm^−2^ during the senescence of the sample leaf in 2022–2023.

## 4. Discussion

### 4.1. Band Assignments

The band maximum position (675 nm) of the broad absorption bands in the 550–750 nm region for the green leaf (Figure 2e) is about 10–15 nm longer than the reported positions around 663 nm for chlorophyll a extracted from green leaves in organic solvents such as diethyl ether and acetone [1,6,46,47] (Figure 1b). This 663 nm band is reported to shift to the longer wavelength (red shift) in the presence of charged polar components [48]. The red shift of chlorophyll a in the plant tissue (in vivo) can originate from the presence of water and the binding of chlorophyll a in the photosynthetic complexes (photosystems).

The broad absorption bands in the 550–750 nm region for the green leaf have shoulders in the 550–650 nm region (Figure 2e). These shoulder bands can include contributions from the 617 nm band of chlorophyll a and from the 646 nm band of chlorophyll b, but can also include the 607 and 665 nm bands of pheophytin a and the 608 and 668 nm bands of pheophorbide a during chlorophyll breakdown [1,6,49] (Figure 1b).

Another broad band in the 400–550 nm region, centered around 490 nm (Figure 2e), can be due mainly to carotenoids such as β-carotene, containing bands around 454 and 480 nm [6,47] (Figure 1c). However, this broad band might include a contribution from chlorophyll b around 460 nm and chlorophyll a around 430 nm [6,47] (Figure 1b). Therefore, this broad band can be due to both carotenoids and chlorophylls. In particular, chlorophyll b contributions can be significant. This will be discussed later.

The band position around 570 nm of the broad band in the 520–650 nm region for the red leaves (Figure 2e) is red-shifted from the reported band position of anthocyanin extracted in organic solvents, which is centered around 546 nm [47]. Anthocyanins in some flowers extracted in water are reported to show absorption maxima around 585–588 nm with shoulders around 542–546 and 505–512 nm [50]. The authors explained that the red shift of the absorption maxima of these anthocyanins, which are complexes with proteins, is due to ionic binding and complexation with proteins. Therefore, the observed red shift of the anthocyanin band can be due to the presence of anthocyanins in protein complexes in water.

### 4.2. Comparison of Changes in Band Areas and Reflectance Indices with Time

To examine quantitative changes in the absorption bands observed over time in the absorption spectra of the sample leaf in 2022–2023, the band areas in the 450–530, 530–600, and 650–700 nm ranges with linear baselines were selected to avoid overlapping bands (Figure 4a). In this section, changes in these band areas with time are compared with reflectance indices often used in the remote sensing of plants [28,29,30,31,32] (Figure 5).

Changes in the Anth red-edge index (mARI) with time for anthocyanins, shown in Figure 5e, are quite similar to those of the band area (530–600 nm) for anthocyanins in Figure 4c. This 530–600 nm region receives only small contributions from the breakdown products of chlorophyll a (chlorophyllide a, pheophorbide a, and pheophytin a) without contributions from chlorophyll a and b or carotenoids. Therefore, this 530–600 nm band can be representative of the content of anthocyanins in senescing Japanese maple leaves. Since this band area is very well correlated to a* values (green/red) in Figure 3e, anthocyanin increases are the main origin of red senescence.

The Car red-edge index for carotenoids increased in the late stage of senescence (Figure 5d), which is not considered reasonable. This can be due to the use of reflectance at 700 nm (red edge) in this index, which is affected greatly by the decreases in chlorophylls (Figure 4a) despite the fact that this index was made for avoiding effects from chlorophylls [30]. On the other hand, the Car green index (CI_green_) decreased slowly until day 40, then rapidly until day 55 (Figure 5c), which is similar to the 450–530 nm band area for carotenoids shown in Figure 4b. This can be understood by the use of reflectance at 510 and 570 nm for this index, representing carotenoids at 510 nm and subtracting the contributions of anthocyanins (Figure 4a). However, it should be noted that while the reflectance index at 510 nm corresponds mainly to carotenoids (Figure 1b,c), the 450–530 nm band area include large contributions from chlorophyll b, but not from chlorophyll a (Figure 1b). Therefore, the 450–530 nm band area represents both carotenoids and chlorophyll b.

The Chl red-edge index (CI_red edge_) decreased slowly from 0 to 40 days, then rapidly until day 60 (Figure 5b). This index is considered to represent the total chlorophyll content [32]. On the other hand, the Chl green index (CI_green_) values were relatively stable until day 40, increased until day 55, then decreased back (Figure 5a). This increase in CI_green_ can be due to the use of reflectance at 550 nm, which is affected by the increase in anthocyanins (Figure 4a). Changes in the band area of 650–700 nm with time for chlorophyll a (Figure 4d) are different from those of the two chlorophyll reflectance indices. This can be understood by the fact that the Chl red-edge index (CI_red edge_) represents the total chlorophyll content, while the Chl green index (CI_green_) can be affected by the increase in anthocyanins. Since the band area of 650–700 nm shows a different behavior from the Chl red-edge index (CI_red edge_) for the total chlorophyll, with only small contributions from anthocyanins, carotenoids, and chlorophyll b (Figure 1b and Figure 4a), this 650–700 nm band area mainly represents chlorophyll a.

Based on the above arguments, although reflectance indices are also effective for representing the total chlorophyll and anthocyanin contents in senescing leaves, the 450–530, 530–600, and 650–700 nm band areas determined in this study provide new information on the sequential changes in the pigments of senescing leaves.

In the early stage of senescence of the Japanese maple leaf (0–45 days), carotenoids and chlorophyll b (450–530 nm band area) and chlorophyll a (650–700 nm band area) decreased gradually (Figure 4b,d). From 40 to 57 days, anthocyanins (530–600 nm band area) increased rapidly (Figure 4c). Then, from 50 to 55 days, the 450–530 nm band area decreased rapidly (Figure 4b) while the 650–700 nm band area increased rapidly (Figure 4d). This can be explained by the decrease in chlorophyll b and the increase in chlorophyll a, facilitated by the conversion of chlorophyll b to a, which is supposed to occur in the first stage of chlorophyll breakdown (Figure 1a). This might be the first natural evidence of the chlorophyll b to a conversion, which could be obtained by the present band area method.

While reflectance indices are widely used for evaluating pigment contents in plant leaves through remote sensing, they might have some limitations for applications concerning detailed sequential changes for some plants. Although further studies are still needed to establish its validity for different plant species, the present band area method provides an alternative approach to assessing temporal changes in pigment contents in the same leaves, especially their kinetic behavior.

### 4.3. Senescence Processes of the Japanese Maple (Acer palmatum, Katsura) (Momiji) Leaf

Based on the above results and discussion, the senescence processes of the Japanese maple (Momiji in Japanese) leaf from 31 October 2022 to 4 January 2023 (65 days) can be summarized as follows.

During the first stage, from 0 to 40 days, slight decreases in chlorophyll a (650–700 nm band area) and chlorophyll b and carotenoids (450–530 nm band area), as well as slight increases in anthocyanins (530–600 nm band area), were observed (Figure 4). These are reflected by the slight decreases in b* values (yellow) and slight increases in a* values (green/red) during the first stage (Figure 3e,f).

In the second stage, from 40 to 50 days, carotenoids and chlorophyll b decreased in association with the b* decrease (Figure 3f and Figure 4b). Anthocyanins increased rapidly, resulting in a rapid increase in the a* value (Figure 3e and Figure 4c).

In the third stage, from 50 to 55 days, anthocyanins and the a* value continued to increase rapidly (Figure 3e and Figure 4c). Carotenoids and chlorophyll b decreased rapidly while chlorophyll a increased rapidly (Figure 4b,d). This represents the conversion of chlorophyll b to chlorophyll a, which is the first step of chlorophyll breakdown [2] (Figure 1a).

The fourth stage, from 55 to 65 days, can be characterized by a very rapid decrease in chlorophyll a followed by a decrease in anthocyanins from day 57 onward (Figure 4c,d), giving the highest reddening of the leaf, with a maximal a* value at day 60 (Figure 3e). From 60 to 65 days, chlorophyll a continued to decrease rapidly with the decrease in anthocyanins (Figure 4c,d), resulting in a decrease in a* values and an increase in b* values, causing brownish colors (Figure 3e,f).

It should be noted that after the increase in anthocyanins from 40 to 57 days, chlorophyll a started to decrease very rapidly from day 55 onward in the maximal reddening period (Figure 3e and Figure 4c,d). The origin of the formation of anthocyanins during the leaf senescence period has been proposed to protect leaf cells losing chlorophylls against solar radiation damage [25,26,27]. The sequential changes in pigment contents obtained in this study support this light screen hypothesis in a logical order. When the formation of anthocyanins starts, chlorophyll breakdown processes first occur through the conversion of chlorophyll b to chlorophyll a (Figure 4b–d). After the formation of enough anthocyanins, the leaf becomes ready to break down chlorophyll a, possibly through enzymes. STAY-GREEN (SGR) proteins have been reported to destabilize chlorophyll–protein complexes, spurring the further degradation of chlorophylls and proteins [2,20,21,22]. Therefore, the rapid chlorophyll a breakdown process can be catalyzed by SGR (Figure 1a, left).

### 4.4. Comparison with Other Series of Senescenece of Japanese Maple (Momiji) Leaves

In order to compare the above data concerning the Japanese maple (Momiji) leaf from 31 October 2022 to 4 January 2023 (65 days) with other series of senescence processes in Japanese maple leaves, two different series were analyzed using the same spectro-colorimetry.

#### 4.4.1. Daily Monitoring of Senescence of a Japanese Maple (Momiji) Leaf in 2021–2022

**Figure 6:**
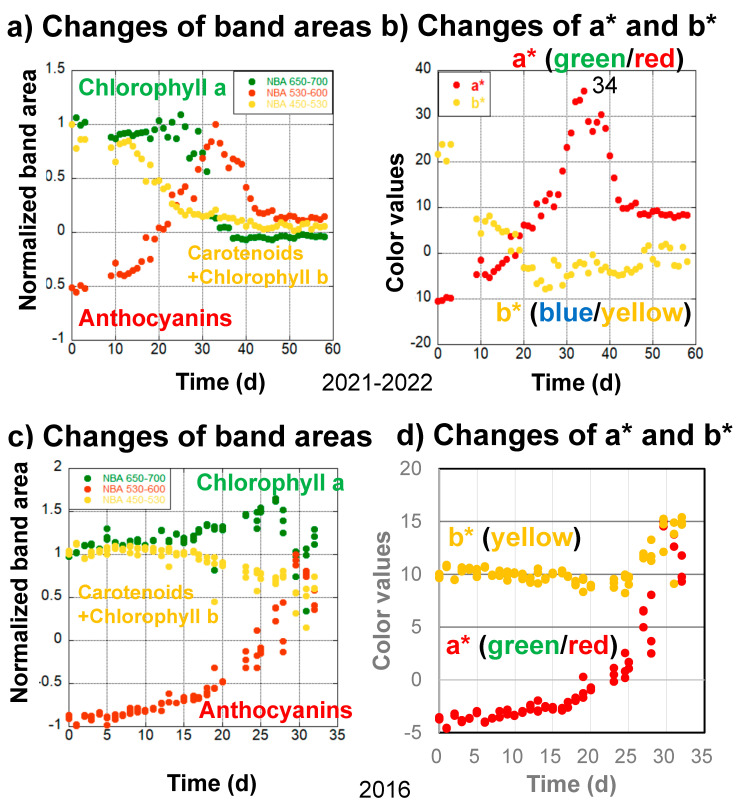
Changes in the (**a**) normalized band areas of chlorophyll a (650–700 nm), carotenoids and chlorophyll b (450–530 nm), and anthocyanins (530–600 nm), as well as (**b**) a* (green/red) and b* (blue/yellow) color values, during the daily monitoring of the senescing processes of a Japanese maple leaf from November 14, 2021 to January 11, 2022 (59 days). Changes in the (**c**) normalized band areas of chlorophyll a (650–700 nm), carotenoids and chlorophyll b (450–530 nm), and anthocyanins (530–600 nm), as well as (**d**) a* (green/red) and b* (blue/yellow) color values, during the daily monitoring of the senescing processes of a Japanese maple leaf (triplicate measurements) from 24 October 2016 to 25 November 2016 (32 days).

The second series of data concerns the daily monitoring of a Japanese maple (Momiji) leaf from 14 November 2021 to 11 January 2022 (59 days) on the veranda of the first author’s home (Figure 6a,b). Data are lacking from the fourth to eighth days because the light source of the spectrometer was being changed. During the first stage, from 0 to 10 days, gradual decreases in chlorophyll a and carotenoids and chlorophyll b, as well as an increase in anthocyanin, can be observed based on the spectral band area analyses (Figure 6a). These are reflected by the decreases in b* values and increases in a* values during the first stage (Figure 6b).

In the second stage, from 10 to 25 days, anthocyanins increased rapidly, resulting in a rapid increase in a* values (Figure 6a,b). Carotenoids and chlorophyll b decreased in close association with decreasing b* values, while chlorophyll a increased (Figure 6a,b).

In the third stage, from 25 to 34 days, anthocyanin and a* value increases continued, reaching maxima around day 34, while carotenoids and chlorophyll b decreased slightly and b* values remained roughly the same. On the other hand, chlorophyll a started to decrease rapidly (Figure 6a). These processes caused the highest reddening in the leaf, with maximal a* values at day 34 (Figure 6b).

The fourth stage, from 34 to 59 days, can be characterized by a very rapid decrease in chlorophyll a from 30 to 40 days as well as a decrease in anthocyanins from 33 to 50 days (Figure 6a).

After the beginning of anthocyanin formation, the conversion of chlorophyll b to chlorophyll a started. When sufficient anthocyanins were formed, a rapid decrease in chlorophyll a began in a similar sequential order as that in the 2022–2023 data (Figure 3, Figure 4, and Figure 6).

#### 4.4.2. Daily Monitoring of Senescence of Japanese Maple (Momiji) Leaves in 2016

The third series of data concerns the daily monitoring (triplicate measurements) of Japanese maple (Momiji) leaves from 24 October 2016 to 25 November 2016 (32 days) by the second author, using one of four selected leaves on a naturally grown maple tree on the campus of Osaka University (Figure 6c,d). The handheld spectrometer used in these measurements was different from the instrument in Figure 2, covering only the wavelength range from 400 to 760 nm [34].

During the first stage, from 0 to 10 days, only slight changes were observed for chlorophyll a, carotenoids and chlorophyll b, anthocyanins, and a* and b* values (Figure 6c,d).

In the second stage, from 10 to 27 days, anthocyanins started to increase, carotenoids and chlorophyll b decreased, and chlorophyll a increased in association with increases in a* values and slight decreases in b* values (Figure 6c,d).

In the third stage, from 27 to 32 days, anthocyanins and a* values continued to increase, while carotenoids and chlorophyll b fluctuated with b* value increases. Chlorophyll a started to decrease rapidly, with some fluctuations (Figure 6c,d). Although the early slow chlorophyll a decrease could not be observed because of the shorter monitoring period, these sequential changes were mostly similar to the 2022–2023 and 2021–2022 data.

### 4.5. Kinetics of Chlorophyll a Decreases in the Japanese Maple (Momiji) Leaves

**Figure 7:**
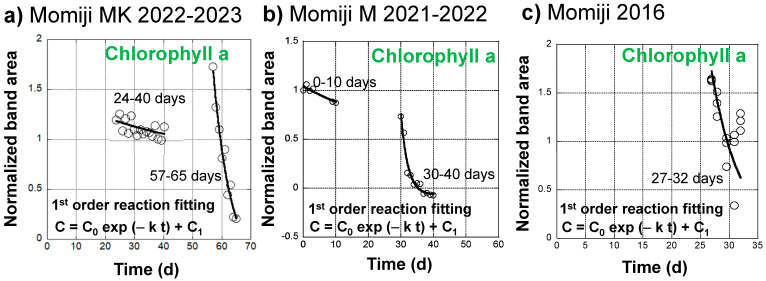
Curve fitting of the normalized band area changes of chlorophyll a (650–700 nm) through a first-order kinetic decrease (C = C_0_ exp (−k t) + C_1_) during the daily monitoring of the senescing processes of Japanese maple leaves. (**a**) A slower decrease from 24 to 40 days and a faster decrease from 57 to 65 days during the 31 October 2022 to 4 January 2023 period; (**b**) A slower decrease from 0 to 10 days and a faster decrease from 30 to 40 days during the 14 November 2021 to 11 January 2022 period; (**c**) A faster decrease from 27 to 32 days during the 24 October 2016 to 25 November 2016 period.

Changes in the normalized band area of chlorophyll a during the senescence of a Japanese maple leaf in 2022–2023 appeared to exhibit two different decreasing trends in the slow earlier and fast later stages (Figure 4d). Therefore, they are replotted in Figure 7a by eliminating the stable period from 0 to 23 days and the increasing period from 40 to 56 days due to the conversion of chlorophyll b to chlorophyll a.

The normalized chlorophyll a band areas from 24 to 40 days were tentatively fitted using the following equation, assuming a first-order decreasing reaction:

C = C_0_ exp (−k t) + C_1_ (16)

The fitting curve is shown in Figure 7a. The fitting parameters are as follows: C_0_ = 0.408, C_1_ = 0.783, k = 0.0227 d^−1^, with a correlation factor of R = 0.589. Although the fitting is not good, the obtained decrease rate constant, k = 2.63 × 10^−7^ s^−1^, is very small. Temperatures in this period ranged from 3.9 to 32.4 °C, with an average of 12.9 °C.

The chlorophyll a band areas from 57 to 65 days were also fitted using the above equation for the first-order decreasing reaction. The fitting curve satisfactorily reproduced the measured data (Figure 7a) with the following fitting parameters: C_0_ = 2.02, C_1_ = −0.322, k = 0.166 d^−1^, with a good correlation factor of R = 0.980. The obtained decrease rate constant, k = 1.92 × 10^−6^ s^−1^, is about one order of magnitude faster than that in the earlier stage. Temperatures in this period ranged from 2.0 to 29.7 °C, with an average of 8.7 °C.

Changes in the normalized band area of chlorophyll a during the senescence of a Japanese maple leaf in 2021–2022 were also fitted using the first-order reaction equation (Equation (16)) for the two stages from 0 to 10 days and from 30 to 40 days (Figure 7b). The fitting parameters are listed in Table 1 together with the temperature data. The chlorophyll a decrease can be separated into a slower process with a rate constant of k = 0.0269 d^−1^ = 3.11 × 10^−7^ s^−1^ at an average temperature of 14.6 °C and a faster process with a rate constant of k = 0.467 d^−1^ = 5.41 × 10^−6^ s^−1^ at an average temperature of 9.2 °C. These rate constants in 2021–2022 are of a similar orders of magnitude as the slower and faster decreases in chlorophyll a in the Japanese maple leaf in 2022–2023, respectively (Table 1).

The chlorophyll a normalized band area decreases during the senescence of a Japanese maple leaf in 2016 were fitted using the first-order reaction equation (Equation (16)) for the fast later stage from 27 to 32 days (Figure 7c). The fitting parameters are listed in Table 1 together with the temperature data. Data scattering by the triplicate measurements gave a rate constant value range of k = 0.3 ± 0.1 d^−1^ = 2.3 ± 1.2 × 10^−6^ s^−1^ at an average temperature of 13.0 °C. These rate constants in 2016 are of a similar order of magnitude as the faster decreases of chlorophyll a in the Japanese maple leaves in 2022–2023 and 2021–2022 (Table 1).

### 4.6. Origins and Kinetics of Chlorophyll a Decrease in Japanese Maple (Momiji) Leaves

The above three series (2022–2023, 2021–2022, 2016) monitoring the senescence processes of Japanese maple leaves using the same spectro-colorimetry showed the following sequential changes in the pigments of the leaves (Figure 3, Figure 4, Figure 5 and Figure 6). (1) In the earlier stage, chlorophyll a started to decrease slowly. (2) When the anthocyanin formation started, conversion of chlorophyll b to chlorophyll a proceeded as the first step of chlorophyll breakdown. (3) After the formation of enough anthocyanins, a rapid decrease in chlorophyll a started. (4) Then, anthocyanins were also decomposed in the final stage of browning before the fall of leaves. As discussed previously, these sequential processes can be understood through the light screen hypothesis on the origin of anthocyanin increases during leaf senescence, which are posited to protect leaf cells losing chlorophylls against solar radiation damage [25,26,27].

**Figure 8:**
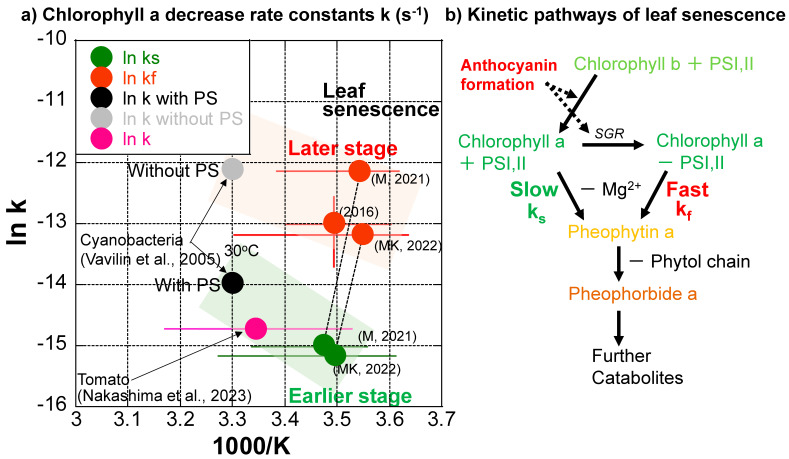
(**a**) Chlorophyll a decrease rate constants of Japanese maple leaves plotted in an Arrhenius diagram for slower rates (k_s_) in earlier stages (green) and faster rates (k_f_) in later stages (red) of leaf senescence periods in 2016, 2021, and 2022, with error bars for temperature ranges. The chlorophyll a decrease rate constant determined for the ripening of a mini tomato in 2022 is plotted with an error bar for the temperature range (purple–pink) [33]. Chlorophyll a decrease rate constants for cyanobacteria with and without photosystems (PS) I and II at 30 °C [51] are also shown (black and gray). (**b**) Tentative model for the kinetic pathways of leaf senescence. In the early stage, the breakdown of chlorophyll a in PSI, II is slow, with the rate constant k_s_. In the middle stage, when anthocyanin formation starts, chlorophyll b is converted to chlorophyll a in PSI, II. In the late stage, when enough anthocyanin is formed to protect the cells against sunlight, STAY-GREEN protein (SGR) decomposes chlorophyll–protein complexes in PSI, II and free chlorophyll a is rapidly broken down, with the fast rate constant k_f_.

The chlorophyll a decreases during the three series of data, except for the slower one in 2016, indicate that they are separated into slower and faster decrease processes with smaller and larger first-order rate constants (Figure 7a–c). Although variations in temperature ranges are quite large during these processes (Table 1), natural logarithms of the rate constant (k (s^−1^) (ln k)) data are tentatively plotted against 1/T (absolute temperature: K) (1000/T for easy conversion to kJ·mol^−1^ dimension) in an Arrhenius diagram in Figure 8a, with corresponding error bars for temperature ranges. The value range of the higher chlorophyll a decrease rate constants in 2016 are also shown as an error bar because of the triplicate measurements. Errors in other rate constants roughly correspond to symbol sizes. The slower decreases in the earlier stage (green points) and the faster decreases in the later stage (red points) of leaf senescence are of similar orders of magnitude, respectively (Figure 8a).

Although the detailed chlorophyll breakdown pathways could not be examined because we could not chemically analyze the same sample multiple times during monitoring, the decrease in greenish color before reddening during leaf senescence suggests the following pathway: Chlorophyll b → Chlorophyll a (green) → Mg^2+^ release from the tetrapyrol ring → Pheophytin a (yellow-brown) → release of phytol chain → Pheopholbide a (brown) (Figure 1a, left side and Figure 8b).

The first author used the same spectro-colorimetry to monitor the ripening process of a mini tomato on the same veranda daily from 22 April 2022 to 13 August 2022 [33]. The chlorophyll a decrease kinetics were examined in the same manner as the present study during the ripening of the mini tomato. Surprisingly, the chlorophyll a decrease throughout the whole tomato ripening period of 100 days could be well fitted by a single first-order reaction, giving a rate constant of k = 3.52 × 10^−2^ d^−1^ = 4.07 × 10^−7^ s^−1^ at an average temperature of 25.9 °C. This data (purple–pink) is plotted in Figure 8a with a corresponding error bar for the temperature range (10.2–41.8 °C) (Table 1). This chlorophyll a decrease rate is close to the slower chlorophyll a decrease rate in the earlier stage of leaf senescence (green) (Figure 8a).

Chlorophyll synthesis and degradation rates have been reported for a cyanobacteria (*Synechocystis* sp. PCC 6803) with and without photosystems (PS) I and II during its incubation at 30 °C [51]. The researchers reported chlorophyll degradation rate constants (k) with PS = 8.61 × 10^−7^ s^−1^ and without PS = 5.61 × 10^−6^ s^−1^ at 30 °C. These are plotted in Figure 8a. Interestingly, the chlorophyll degradation rate constant of cyanobacteria with photosystems (wild type) (black) is close to that during tomato ripening (purple–pink) and those at earlier slower stages of leaf senescence (green) (Figure 8a). On the other hand, the chlorophyll degradation rate constant (k) of cyanobacteria without photosystems I and II (gray) is close to those in later, faster stages of leaf senescence (red) (Figure 8a).

Therefore, the slower chlorophyll a decreases in the earlier stages of leaf senescence (green points in Figure 8a) and during tomato ripening (purple–pink point in Figure 8) might correspond to natural chlorophyll degradation processes protected by photosystems including proteins and membranes. On the other hand, the faster chlorophyll a decreases in later stages of leaf senescence (red points in Figure 8a) might correspond to chlorophyll degradation processes without protections by proteins and membranes in photosystems (Figure 8b). Although most enzymes facilitating chlorophyll breakdown have been identified in leaf senescence [19], their activities in different natural systems need further study. In the senescence of Japanese maple leaves, enzymes contributing to different steps of chlorophyl breakdown and anthocyanin synthesis are not well documented. Therefore, details of enzymatic contributions to chlorophyll breakdown rates are not certain. Alternative tentative explanations in the present study include the following: (I) During the earlier stage of the leaf senescence period, chlorophyll a decreases slowly, keeping the photosystems and being protected by proteins. (II) When anthocyanins are sufficiently formed for protecting cells against solar radiation, photosystems might be broken, and chlorophyll a decreases rapidly without the protection of proteins in photosystems (Figure 8b).

It should be noted that the chlorophyll decrease rates in natural systems have never been quantitatively reported, to the author’s knowledge, except for our recent data concerning mini tomato ripening [32]. Although further detailed studies are needed, the present data on the chlorophyll decrease rates during three series of senescence of Japanese maple leaves provide some of the first natural evidence of chlorophyll degradation rates.

## 5. Conclusions

Leaf senescence processes of Japanese maple (*Acer palmatum*) were followed daily in the same positions for approximately 30–60 days using visible spectroscopy with two original handheld visible spectrometers during three autumn–winter seasons (2016, 2021, and 2022). The obtained reflection spectra were used to calculate reflectance indices, as proposed by the literature. They were also converted to absorption spectra and band areas of chlorophyll a (650–700 nm), carotenoids and chlorophyll b (450–530 nm), and anthocyanins (530–600 nm) were determined. Changes in reflectance indices, color values (L*, a*, b*), and normalized band areas with time were analyzed.

1. The chlorophyll red-edge index (CI_red edge_) decreased during the leaf senescence, corresponding to the total chlorophyll contents.
2. The chlorophyll green index (CI_green_) increased in the late stage of leaf senescence, possibly due to the use of reflectance at 550 nm being affected by the increase in anthocyanins.
3. The carotenoid red-edge index (Car_red edge_) increased during the leaf senescence, possibly due to the use of reflectance at 700 nm being affected by the decrease in chlorophylls.
4. The carotenoid green index (Car_green_) decreased during the leaf senescence, similar to the 450–530 nm band area caused by carotenoids and chlorophyll b.
5. The anthocyanin red-edge index (mARI) increased during the leaf senescence, similar to the 530–600 nm band area caused by anthocyanins.
6. The 650–700 nm band area determined in this study showed different changes with time in CI_red edge_ and CI_green_, and was considered to represent chlorophyll a.
7. Therefore, the band areas newly determined in this study provide new information for changes in chlorophyll a (650–700 nm band area), chlorophyll b (and carotenoids) (450–530 nm), and anthocyanins (530–600 nm) with time.
8. The a* value (green/red) increased slowly in the early stage (decrease in green color) associated with slow decreases in the chlorophyll a band area.
9. The b* value (yellow) decreased slowly in the early stage (decrease in yellow color) associated with decreases in the carotenoid and chlorophyll b band area.
10. In the middle stage, the carotenoid and chlorophyll b band area decreased and the chlorophyll a band area increased. This can be explained by the conversion of chlorophyll b to chlorophyll a as a preparation for further chlorophyll breakdown.
11. In the later stage, the a* value rapidly increased parallel to the anthocyanin band area.
12. In this later stage, after the anthocyanin band area increased sufficiently, a rapid decrease in the chlorophyll a band area started.
13. These sequential daily changes monitored by the present method support the light screen hypothesis of the formation of anthocyanin for the protection of leaf cells losing chlorophylls against solar radiation damage.
14. Decreases in the chlorophyll a band area with time could be fitted by first-order kinetics, resulting in the earlier slow and the later fast decrease rate constants of k_s_ = 2.6 ~ 3.1 × 10^−7^ s^−1^ and k_f_ = 1.9 − 5.8 × 10^−6^ s^−1^, respectively, providing some of the first kinetic data on natural chlorophyll breakdown processes.
15. The slower rate constants (k_s_) are close to that reported for chlorophyll a decreases in the ripening of a mini tomato [33].
16. The slower rate constant (k_s_) is also close to the chlorophyll a decrease rate of cyanobacteria (PCC6803) with photosystems (wild type), while the faster rate (k_f_) is close to that of cyanobacteria without photosystems I+II (mutant) [51].
17. Although detailed reaction pathways could not be examined because of the impossibility of conducting multiple chemical analyses of the same samples, the slow and fast chlorophyll breakdown can be tentatively explained by the presence or absence of protection of chlorophylls by proteins in photosystems.
18. The present spectroscopic method based on absorption band areas can provide an alternative method for evaluating pigment contents in plants, especially for their kinetic behavior, but further studies are needed to examine the validity for different plant species.

## Figures and Tables

**Table 1:** Year, periods (days), maximum, average, minimum temperatures (°C), average absolute temperatures T (K), fitting parameters (C_0_ and C_1_), correlation coefficients (R) of the first-order decrease kinetics (C = C_0_ exp (−k t) + C_1_), and obtained slower and faster chlorophyll decrease rate constants (k_s_ (s^−1^) and k_f_ (s^−1^)) and their natural logarithms.

Chl a Decrease Rates	Period	Tmax	T (°C)	Tmin	T (K)	1000/T	C_0_	C_1_	R	k_s_ (s^−1^)	k_f_ (s^−1^)	ln k_s_	ln k_f_
MK, 2022	1: 24–40d	32.4	12.9	3.9	286.05	3.496	0.408	0.783	0.589	2.6 × 10^−7^		−15.2	
MK, 2022	2: 57–65d	29.7	8.7	2.0	281.85	3.548	2.020	0.322	0.980		1.9 × 10^−6^		−13.2
M, 2021	1: 0–10d	27.2	14.6	8.2	287.75	3.475	0.681	0.361	0.924	3.1 × 10^−7^		−15.0	
M, 2021	2: 30–40d	22.3	9.2	2.7	282.35	3.542	0.803	−0.047	0.982		5.4 × 10^−6^		−12.1
M, 2016	2: 27–32d	20.8	13.0	4.2	286.15	3.495	1.700	0.000	0.424		2.3 × 10^−6^		−13.0
Tomato, 2022 *	0–100d	41.8	25.9	10.2	299.05	3.344	8.190	−0.590	0.988	4.1 × 10^−7^		−14.7	
Synechocystis **	With PS		30.0		303.15	3.299				8.6 × 10^−7^		−14.0	
PCC6803 **	Without PS		30.0		303.15	3.299					5.6 × 10^−6^		−12.1

* (Nakashima et al., 2023) [33]; ** (Vavilin et al., 2005) [51].

## Data Availability

All the data are presented in the present paper.
